# Supplementary figures and images for: Age-Related microRNA Overexpression in Lafora Disease Male Mice Provides Links between Neuroinflammation and Oxidative Stress
Source: Int J Mol Sci. 2023 Jan 6;24(2):1089. doi: 10.3390/ijms24021089 (PMC9865572; doi:10.3390/ijms24021089)

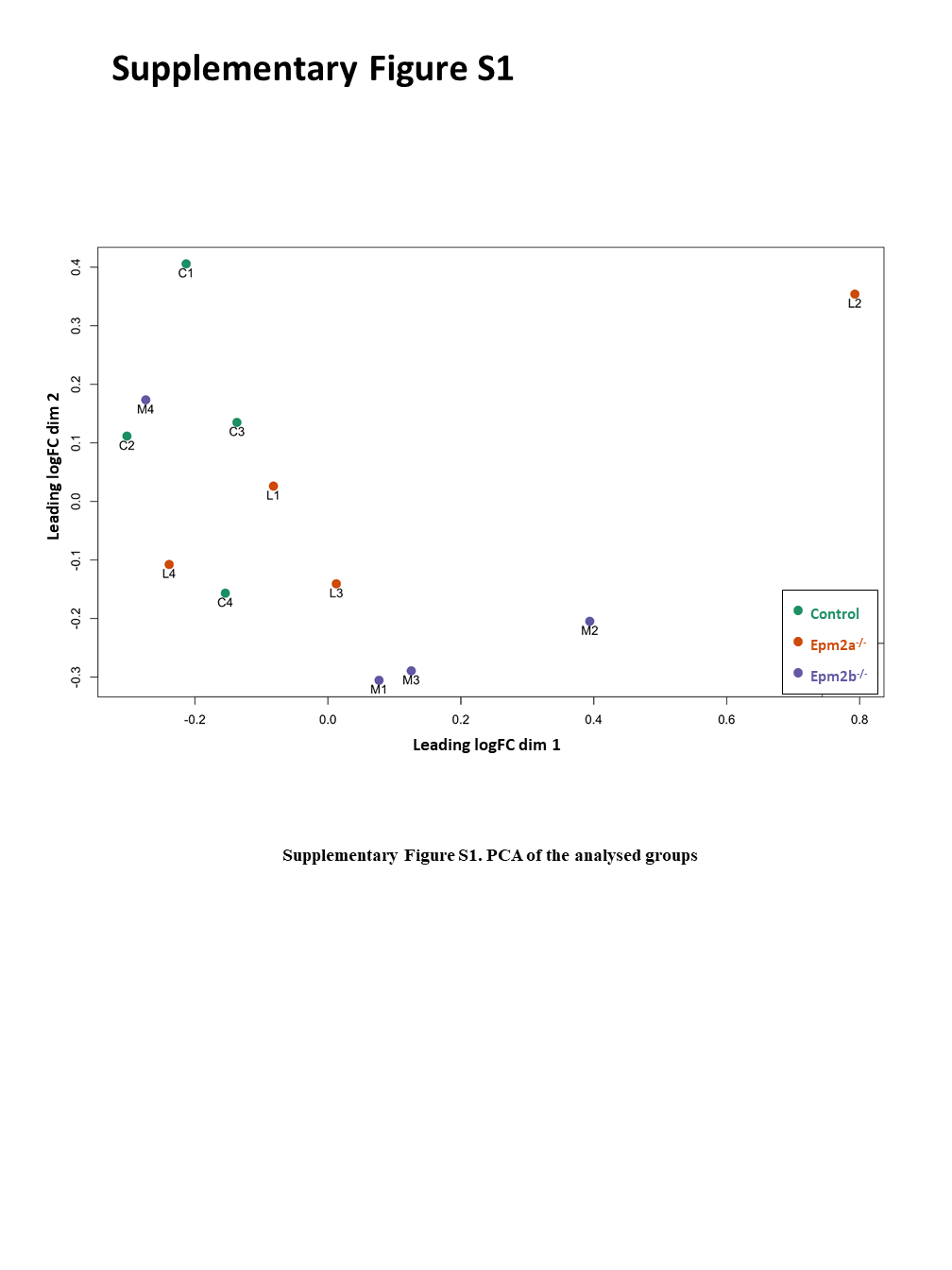

Supplement: Supplementary file 1 [file ijms-24-01089-s001.zip › SupFigureS1.TIF]

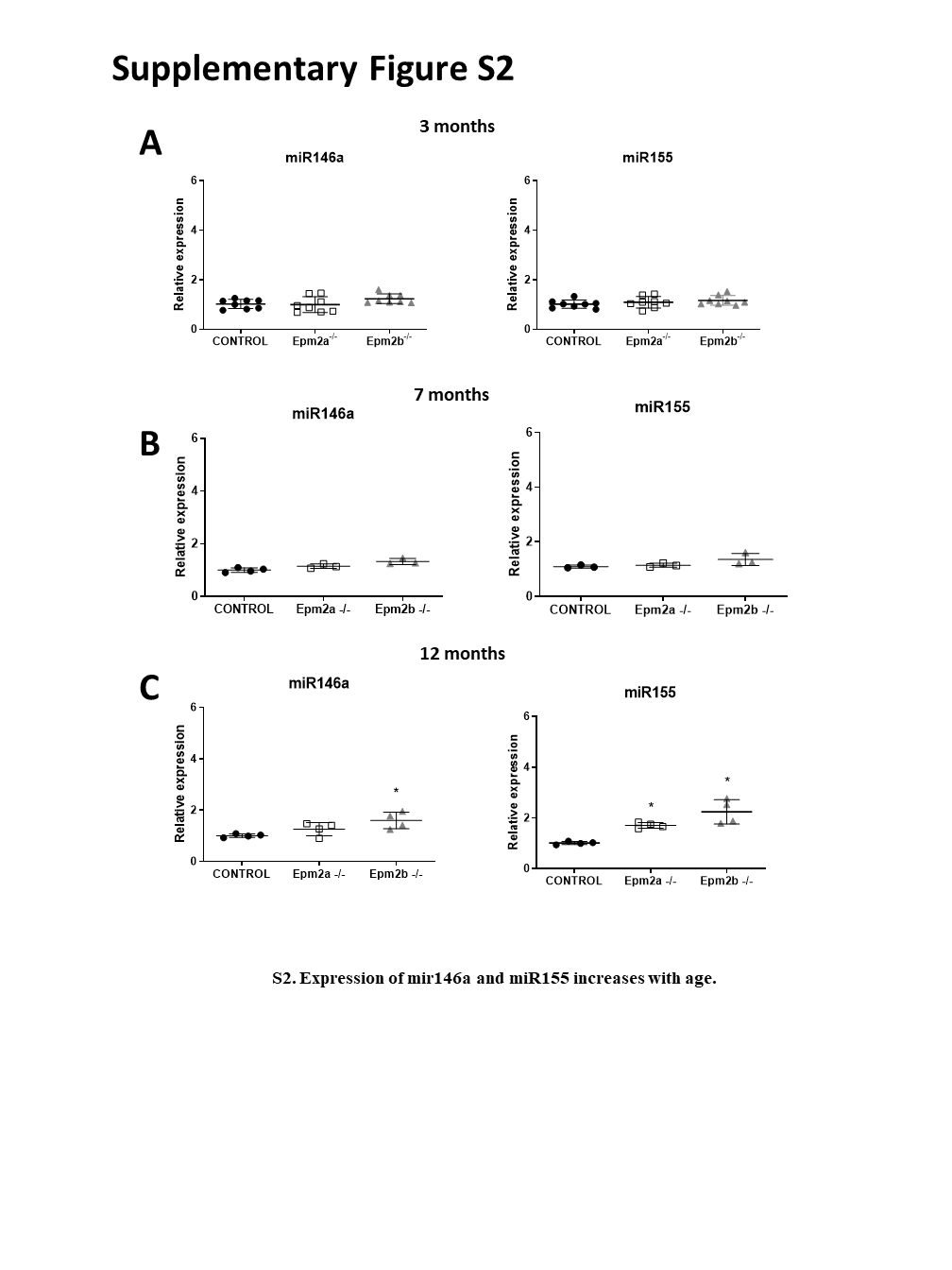

Supplement: Supplementary file 1 [file ijms-24-01089-s001.zip › SupFigureS2.TIF]
